# Hyaluronan derivative HYMOVIS® increases cartilage volume and type ii collagen turnover in osteoarhritic knee: data from MOKHA study

**DOI:** 10.1186/s12891-019-2667-0

**Published:** 2019-06-18

**Authors:** Yves Henrotin, Raveendhara Bannuru, Michel Malaise, Hang-korng Ea, Cyrille Confavreux, Jacques Bentin, Didier Urbin-Choffray, Thierry Conrozier, Jean-Pierre Brasseur, Philippe Thomas, Anne-Christine Hick, Alessandro Marinello, Nicola Giordan, Pascal Richette

**Affiliations:** 1 Bone and Cartilage Research Unit, Arthropole Liège, Université de Liège, Institute of Pathology, CHU Sart-Tilman, 4000 Liège, Belgium; 2 Artialis SA, GIGA Tower, CHU Sart-Tilman, Liège, Belgium; 3 Center for Treatment Comparison and Integrative Analysis, Division of Rheumatology, Tufts Medical Center, Boston, MA USA; 4 Service de Rhumatologie - CHU Sart-Tilman, Liège, Belgium; 5 Service de Rhumatologie - Centre Viggo Petersen, Hôpital Lariboisière, Paris, France; 6 Department of Rheumatology, Hospices Civils de Lyon, Lyon, France; 7 Service de Rhumatologie - CHU Brugmann, Bruxelles, Belgium; 8 Service de Médecine Physique - CHR Citadelle, Liège, Belgium; 9 Service de Rhumatologie - Hôpital Nord Franche-Comté, Trévenans, France; 10 Service de Rhumatologie - CHU UCL Namur - Site de Godinne, Yvoir, Belgium; 11 Service de Rhumatologie - CHR Metz-Thionville - Hôpital Bel Air, Thionville, France; 12 Fidia Farmaceutici, S.p.A, Abano Terme, Italy

**Keywords:** Hyaluronic acid, Osteoarthritis, Cartilage, Biomarkers

## Abstract

**Background:**

The objective of this pilot study was to identify biological, clinical or structural biomarkers of an intra-articular hyaluronic acid injection efficacy (HYMOVIS®) for the design of a larger placebo-controlled clinical trial studying the disease-modifying activity of this treatment.

**Methods:**

Forty six patients with symptomatic knee Osteoarthritis (OA) were enrolled in this open-label, prospective, multicenter, pilot study. Patients received two treatment cycles of intra-articular injections (3 mL) of HYMOVIS® (8 mg/mL of hyaluronic acid hexadecylamide) at 6 months interval. Each treatment cycle involved two intra-articular injections 1 week apart. All patients had Magnetic Resonance Imaging (MRI) of the target knee at baseline and 1 year, and blood samples to assess joint biomarkers. The primary outcome was the change in type II collagen-specific biomarkers (Coll2–1, Coll2–1NO2 and CTX-II) after HYMOVIS® treatment versus baseline. Secondary endpoints included levels changes in aggrecan chondroitin sulfate 846 epitope (CS-846), Cartilage Oligomeric Matrix Protein (COMP), procollagen type II N-terminal propeptide (PIIANP), Matrix Metalloprotease (MMP)-3, Myeloperoxidase (MPO) and Interleukin (IL)-6 serum biomarkers, the ratio Coll2–1/PIIANP, CTX-II/PIIANP, variation of MRI cartilage volume, and Knee injury and Osteoarthritis Outcome Score (KOOS) index.

**Results:**

Coll2–1 serum levels significantly increased overtime while Coll2–1NO2 levels were only increased at D360. Serum PIIANP levels also progressively and significantly enhanced with time. In contrast, other serum biomarker levels including CTX-II, CS-846, COMP, MMP-3, MPO or IL-6 did not change significantly overtime. Interestingly, the ratios Coll2–1/PIIANP and CTX-II/PIIANP decreased, indicating a decrease of cartilage catabolism. Compared to baseline value, MRI cartilage volume and thickness increased in lateral femoral and lateral trochlea compartments and not in medial compartment. These results, in addition to an improvement of T2 mapping score suggest a positive structural effect of the product. Interestingly, WORMS effusion score, an indicator of synovitis, significantly decreased. Finally, global KOOS score and subscales significantly increased overtime while pain at rest, walking pain and patients or investigators global assessment of disease activity decreased. The safety profile was favorable with a low incidence of injection-site pain.

**Conclusion:**

HYMOVIS®, a well-tolerated intra-articular treatment, significantly enhanced type II collagen turnover as suggested by the increase in Coll2–1 and PIIANP levels and cartilage volume observed by MRI in lateral knee compartment. Importantly, this study provides critical information for the design of a larger phase III clinical trial investigating Disease Modifying effect of HYMOVIS®.

**Trial registration:**

http://www.isrctn.com/ISRCTN12227846 11/02/2015.

## Background

Osteoarthritis (OA) is a disorder involving movable joints characterized by cartilage degradation initiated by micro- and macro-injury, synovial membrane inflammation and abnormal subchondral bone remodeling. The disease manifests first as a molecular derangement followed by anatomic, and/or physiologic derangements that can culminate in illness [1]. The knee is the most affected joint by OA. In United States, it has a high prevalence of 40% for men and 47% for women [2].

Up to date, there is no curative treatment for knee OA despite availability of a large number of therapeutic options, including nonpharmacological, pharmacological and surgical therapies. The aim of the pharmacological treatment remains symptomatic to relieve pain and restore function [3–8]. The first-line pharmacological therapy is the use of analgesics such as paracetamol (acetaminophen) up to 4 g per day. However, paracetamol has a short-term analgesic effect and some meta-analysis indicated the occurrence of adverse events i.e. liver injury [9, 10]. The second line therapy is the use of non-steroidal anti-inflammatory drugs (NSAIDs). However, chronic use of NSAIDs can result in serious complications e.g. gastrointestinal bleeding, renal failure, coronary heart disease even at normal dosage [11–13].

Viscosupplementation is recommended in the management of symptomatic knee OA, for appropriate patients, by many scholarly societies of rheumatology and orthopaedics [4, 14–16] sport medicine [17] and geriatrics [18], on the basis of recent systematic reviews and metaanalyses [19–25]. Even though the clinical efficacy is now proved, at least in selected patients, the structure-modifying effect remains to be demonstrated. Two recent studies have suggested that repeat intra-articular (IA) injections of hyaluronic acid (HA) may delay the time to prosthetic replacement [26, 27]. Total knee replacement (TKR) is a valuable surrogate marker of severe OA and a possible endpoint for clinical trials, but unfortunately, is neither a reliable marker of the lack of treatment efficacy nor of the anatomical progression of the disease. Indeed, TKR is highly dependent on intrinsic problems such as access barriers due to geographical and financial considerations, the availability of the resources for TKR and in the willingness of patients to be operated [28]. Therefore, additionnal studies are required to demonstrate the structure-modifying effect HA viscosupplmentation in knee OA.

HYMOVIS® is a sterile, non-pyrogenic, viscoelastic hydrogel for intra-articular injection. The principal component is HYADD®, a novel linear (i.e., not cross-linked) HA chemical derivative containing between 1250 to 1800 disaccharides with a molecular weight comprised between 500 to 730 kDa, displaying unique rheological properties. Indeed, the modification with hexadecylamine creates a network stabilized by reversible hydrophilic and hydrophobic interactions (not by rigid covalent cross-links), conferring high viscoelasticity to this HA derivative. Unlike rigid chemically cross-linked HAs, the reversible interactions stabilizing the mobile reticulum allow for a complete recovery of the 3D structure of the gel (and, therefore, of its elasticity) after mechanical shocks [29]. Thus, hexadecylamine structure improves shock absorbing function of synovial fluid, and potentially protect cartilage and soft tissues against mechanical injuries. These properties together with the prolonged residence time (between 2 to 5 weeks in rodents) in the articular joints enable HYMOVIS® to relieve pain and to improve joint function with a short treatment regimen. In a recent preclinical study [30], HYMOVIS® has exhibited beneficial effects on both chondrocyte and synovial fibroblast expression of catabolic enzymes and inflammatory cytokines/mediator. Recently, a retrospective study and one open-label study have reported that two intra-articular injections of HYMOVIS® 1 week apart reduced pain and improved function for at least 1 year after the first injection in knee OA patients [31, 32] and one single-center single-blind prospective randomized clinical trial evidenced that two injections 7 days apart of HYMOVIS® provided better short-term (at 26 weeks but not 52 weeks) effects on pain and function than two injections of methylprednisolone acetate in patients with mild to moderate knee osteoarthritis [33].

This pilot study aimed to explore the potential structure-modifying effect of HYMOVIS® in patients suffering of knee OA using a combination of scientifically sound, objective measurements of clinical, biological and MRI-based imaging markers [34].

## Patients and methods

### Study design

**Fig. 1:**
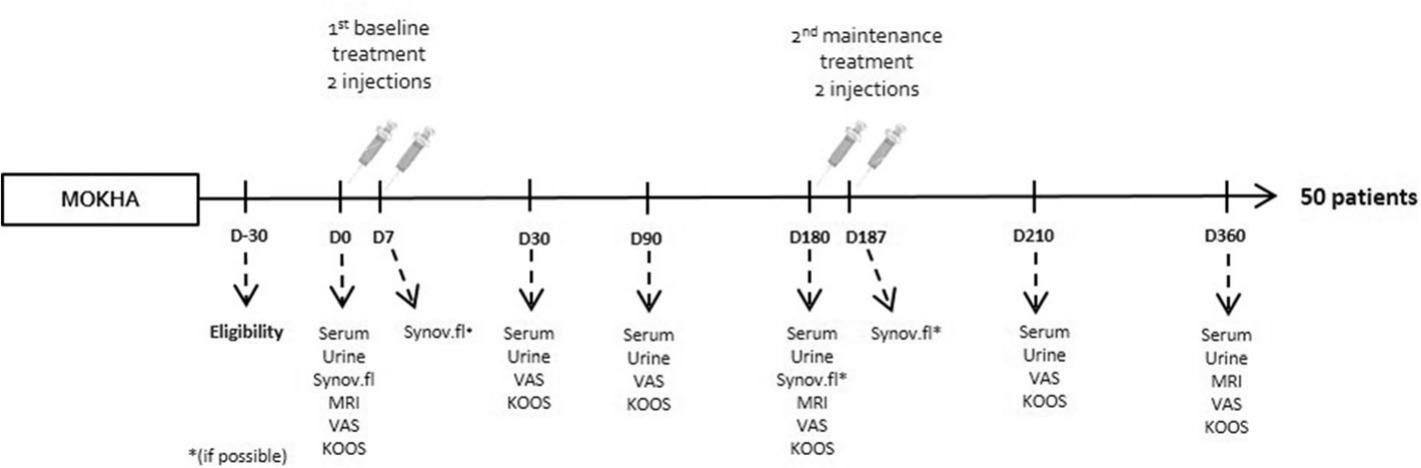
Study design. KOOS = Knee Injury and Osteoarthritis Outcome Score; VAS = Visual Analog Scale; MRI = Magnetic Resonnance Imaging

This was an open, multicenter, prospective study, assessing the effectiveness of two treatment cycles of HYMOVIS® (FIDIA Farmaceutici, Via Ponte della Fabbrica, 3/A 35031 Abano Terme (Padova) Italy; 8 mg/ml of hyaluronic acid partial hexadecylamide in 3 ml sterile syringe [CE0459]) at 6-month interval, each treatment cycle involved 2 intra-articular injections given at 1-week interval (Fig. 1). The study was conducted prospectively by 8 investigators (rheumatologists and rehabilitation medicine physicians) located in Belgium (*n* = 4) and in France (*n* = 4) from public or academic hospitals between February 9th 2015 to June 6th 2017. The same injector had to perform all the injections of a patient to reach homogeneity.

This trial has been conducted according to the “Declaration of Helsinki” and in compliance with Good Clinical Practice (GCP) principles for Medical Devices (ISO14155:2011). Centralized biomarker platform operated under Good Clinical Laboratory Practice guidelines (GCLP/WHO). In Belgium, the protocol has been submitted to the central Ethic Committee (EC) from the University Hospital of Liege and appropriate local ethical committee on September 9th 2014 and approved by November 12th 2014 (B707201422130–2014/247). In France, the protocol has been submitted to the Comité de Protection des Personnes Iles-de France IV and to the French competent authorities (ANSM) as Biomedical Research on February 25th 2015. Approval has been obtained on April 10th 2015 (2015/11) and May 6th 2015(2015-A00370–49), respectively. The study was conducted in strict accordance with the declaration of Helsinki and GCP principles. Each patient received and signed an informed consent.

### Eligibility of patients

Eligible patients were men or women, aged between 40 and 80 years with a Body Mass Index (BMI) ≤ 40 kg/m^2^ suffering of unilateral symptomatic femorotibial knee OA associated or not with femoropatellar knee OA responding to clinical and radiological criteria of ACR (American College of Rheumatology). OA must have been symptomatic for more than 6 months in the most painful knee with a mean global pain at rest determined on Visual Analog Scale (VAS) for the last 24 h over 40 mm (with a washout period for paracetamol and oral NSAIDs depending on the half-life of the drug). Kellgren and Lawrence (KL) score evidenced with X-rays over the past 12 months must have been II or III. Patients signed their informed consent after receiving comprehensive information.

### Exclusion criteria

The exclusion criteria were selected to avoid the presence of a contraindication to treatment or diseases affecting biomarkers clearance and to exclude the interference of concomitant painful condition or therapies that may modulate cartilage metabolism. They also considered contraindications to perform Magnetic Resonance Imaging (MRI). Patients meeting to at least one of the criteria detailed in Table 1 were not included in the study.

**Table 1:** Exclusion criteria

Related to the OA pathology	
• Bilateral (except asymptomatic and grade I) OA of the knee • Radiological K&L grade I or IV • Chondromatosis or villonodular synovitis of the knee • Recent trauma (< 1 month) of the knee responsible of the symptomatic knee • Acute inflammatory osteoarthritis (Kofus ≥7) • Articular disease resulting from articular dysplasia, aseptic osteonecrosis, acromegaly, Paget’s disease, hemophilia, hemochromatosis…. • Inflammatory disease i.e. rheumatoid arthritis, gout and infectious arthritis, acute calcium pyrophosphate arthritis • Pathologies interfering with the evaluation of OA (radiculalgia in the lower limbs, arteritis…..) • Presence of another joint (other than the target knee) affected by OA (confirmed in radiographs and symptomatic)	
Related to treatments	
• Corticosteroids injection in the target knee in the last month before first injection • Hyaluronan injection in the target knee in the last 6 months before first injection • Arthroscopy and surgery in the target knee in the last 6 months before first injection • Oral corticotherapy ≥5 mg/day (in Prednisone equivalent) in the last 3 months before first injection • OA treatments based on curcuma extract (e.g. FLEXOFYTOL) in the last 3 months before first injection • Change in the dosage of symptomatic slow-acting drugs (SYSAD) i.e. chondroitin, glucosamine, diacerein or avocado-soya unsaponifiables in the last 3 months before first injection • Paracetamol and oral NSAIDs before inclusion and follow-up visits (washout period depending on the half-life of the drug). • Osteoporosis-related treatments based on strontium ranelate, selective estrogen-receptor modulator (SERM) and parathormone (PTH) in the last 12 months before first injection • An anticipated need for any forbidden OA treatments during the trial • Contraindications to HYMOVIS®: hypersensitivity to the product components and infections or skin diseases in the area of the injection site • Non-pharmacologic therapy (including physical therapy) for the lower extremities initiated in the month before first injection • Anticoagulant (coumarinic compound) and heparin	
Related to associated diseases	
• Severe diseases (liver or renal failure, lung/heart disease, tumor, HIV….) • Allergy or contra-indication to hyaluronan and constituents • Severe alteration of mobility unabling functional evaluation • High risk of hemorrhage and risk of infection at the site of injection • Anticipated need for any surgical or other invasive procedure during the trial including prosthesis in the target knee	
Related to patients	
• Participation to a therapeutic clinical trial in the last 3 months before first injection • Under guardianship or judicial protection • Pregnancy, breastfeeding, planned conception, premenopausal women without contraception, tubal ligation or hysterectomy	
Related to MRI counter-indication	
• Patient with a pacemaker, an implantable defibrillator, neurosurgical clips, a neurostimulator, cochlear implant, a stent from less than 3 weeks, an insulin pump • Patient with a ferromagnetic splinter in the body, or having wire sutures • Serious mobility problem (Parkinson, tremors), • Claustrophobia	

### Prohibited/authorized treatments

Authorized treatments during the trial were paracetamol only if needed at the maximal dose of 4 g per day and oral NSAIDs only if paracetamol at 4 g per day was not sufficient and only for a period as shorter as possible. Symptomatic Slow Acting Drugs in OA (SYSADOA) including chondroitin, diacerein, glucosamine, soy and avocado unsaponifiables were also authorized in stable dosage and only if began and stable from 3 months before first injection. Similarly, non-pharmacologic modalities (including physical therapy) for the lower extremities were accepted only if it began at least 1 month before first injection and was stable during the trial. Usual treatments taken by the subject in other no OA-related diseases were kept as constant as possible during the trial. Bisphosphonates in stable dosage and only if began for more than 14 days before first injection was also authorized. Prohibited treatments during the study were corticosteroids or hyaluronan injection in any joint, oral corticotherapy, curcumin based treatment (e.g. Flexofytol®), analgesics except paracetamol, NSAIDs at the exception of oral form, anticoagulant (coumarinic compound) and heparin and osteoporosis-related treatments based on strontium ranelate, selective estrogen-receptor modulator (SERM) and parathormone (PTH).

### Outcomes measures

Demographic data, medical history, eligibility criteria, concomitant treatments were recorded at screening visit scheduled 30 days (D30) before the first injection regimen. MRI acquisition was performed in the month preceeding the first injection, but also 6 months (D180) and 12 months (D360) after this injection. Images were transferred to the centralized imaging platform (ARTIALIS SA, Liège, Belgium) for quality check and reading. The semi-quantitative Whole-Organ Magnetic Resonance Imaging Score (WORMS) and its 14 features (articular cartilage integrity, subarticular bone marrow abnormality, subarticular cysts, subarticular bone attrition, marginal osteophytes, medial and lateral meniscal integrity, anterior and posterior cruciate ligament integrity, medial and lateral collateral ligament integrity, synovitis/effusion, intraarticular loose bodies, and periarticular cysts/bursitis) were evaluated in 14 subregions (patella/femur/tibia) of the knee [35]. T2 relaxation time were evaluated in patella, femur and tibia subregions and in the following cartilage sub-regions medial tibia, medial weight bearing femur, medial trochlea, lateral tibia, lateral weight bearing femur and lateral trochlea. Cartilage volume (mm^3^), thickness (mm), and bone curvature were reported for femur, tibia, patella, and for the following cartilage subregions: medial tibia, medial weight bearing femur, medial trochlea, lateral tibia, lateral weight bearing femur and lateral trochlea.

The Knee Injury and Osteoarthritis Outcome Score (KOOS) and its subscale scores using a self-administered questionnaire, the mean knee pain over the last 24 h at rest and while walking using a VAS, the global assessment of disease activity (by patient and by investigator) using a VAS, responder rate to treatment (following OARSI OMERACT criteria), patient satisfaction by a five-category scale (i.e. better, little better, same, little lower or far lower), concomitant treatments, adverse events and drop-off were recorded at each visit. Adverse events were recorded immediately after the injections and during the follow-up visits.

Serum (s) and urine (u) were collected at D0, D30, D90, D180, D210 and D360 for each subject by the clinical laboratory of each investigation site and shipped to the centralized biomarker platform (Artialis SA, Liège, Belgium) for biomarker testing at appropriate frequency. Samples have been assayed in pooled test series using validated immunoassay assays and according to written procedures provided by the manufacturer. sColl2–1 (Artialis SA, Liège Belgium), sColl2–1NO2 (Artialis SA, Liège Belgium), uCTXII (Immunodiagnostic Systems Limited (IDS), Boldon, Tyne and Wear, UK), sCS-846 (IBEX, Montréal, Canada), sCOMP (BioVendor, Brno, Czech Republic), sPIIANP (Merck, Darmstadt, Germany), and sMMP-3, sMPO, sIL-6 (Bio-Techne, Abingdon, UK). CTX-II was normalized on creatinine (Quidel Corporation, San Diego, USA).

#### Primary outcomes

This is a post-marketing study designed to explore the effect of HYMOVIS on cartilage metabolism and to explore the potential of Coll2–1, Coll2–1NO2 and CTX-II as companion biomarkers for the follow-up of patients treated with HYMOVIS. This was the reason for which we have selected Coll2–1, Coll2–1NO2 and CTX-II as primary end-points. The primary outcome measures were the levels of sColl2–1, sColl2–1NO2 and uCTX-II biomarkers at baseline (D0) and at the different time points.

#### Secondary outcomes

The secondary outcome measures were the levels of sCS-846, sCOMP, sPIIANP, sMMP-3, sMPO and sIL-6 biomarkers; the measure of MRI WORMS score, T2 relaxation time (whole, top, middle and bottom layers), cartilage volume, thickness and curvature; function evaluated with KOOS index, VAS for the mean knee pain over the last 24 h at rest and while walking, VAS for the global assessment of disease activity (by patient and by investigator), responder rate to treatment (OARSI-OMERACT criteria), adverse events, drop-off and patient satisfaction scale.

### Statistical analysis

#### Sample size

The sample size was calculated following recommendations and guidance on statistical principles for clinical trials [36, 37], considering a minimal biomarker difference between time points at least equal to the variability of the assay (primary endpoint, s = θ = 10%). It was calculated to estimate a sample size for a future trial studying the disease-modifying effect of the product. Sample size was calculated according to the paired t-test formula assuming a type I error rate of 0.05 and an 80% power (type II error). The sample size varied according to the assumed correlation (Corr) between the pre and post visits. The sample was sized on the worst case (10% correlation). With 17 cases, the power was 90.5, 97.2 and 99.9% in case of higher correlations, respectively 0.3, 0.5 and 0.7. Moreover, the sample size was inflated according to the theoretical responder rate (RR) determined from the literature [38] (RR = 56.8%) and potential study drop-off (DO = 40%). Therefore, a total number of 50 patients was planned to be enrolled.

#### Analysis of the primary outcome

Analyses were performed using the Statistical Analysis Software SAS (version 9.4, SAS Institute Inc., Cary, NC, USA). First, baseline (D0) sColl2–1, sColl2–1 NO_2_ and uCTX-II concentrations were compared with those obtained at D30, D90, D180, D210 and D360 days by using a paired t-test (or a Wilcoxon signed-rank test if the data were deemed non-normal). Moreover, a repeated measures mixed model was applied using the change from baseline in biomarker levels as the outcome variable, visit as qualitative independent variable and baseline x visit interaction as covariate. *P*-values of the fixed effects and covariates have been presented and estimates at each time point have been given with 95% confidence intervals. Analyses were performed both in the Full Analysis Set (FAS) and Per-Protocol (PP) populations. Full Analysis Set (FAS) corresponded to all subjects who received at least one treatment cycle consisting of two injections at 1 week interval. Per-Protocol Set (PP) were all subjects who received all the injections and had no major protocol deviations.

#### Analysis of the secondary outcomes

The secondary biomarkers endpoints were analyzed using the same statistical methods that described for the primary endpoints. The MRI endpoints (WORMS, T2 relaxation time, cartilage volume, thickness and curvature) have been analyzed with the Paired t-test or Wilcoxon signed-rank tests comparing the results observed at D180 and D360 versus the baseline visit (D0). The WORMS values have been analyzed by compartments and using the total score. The T2 relaxation time have been analyzed by layers and using the total score as well. The KOOS (sub-score and total score) and VAS scales have been analyzed using the same statistical methods that biomarkers endpoints. The responder rate according to the OARSI-OMERACT ratio was calculated at each time point and all the analyses of biomarkers has been repeated in subgroups of responders and non-responders. Moreover, the level of biomarkers at each assessment has been compared between responders and non-responders with the T-test (or the Wilcox rank-sum test if the data are deemed non-normal). Correlations between variables (biological and/or MRI-based imaging markers) has been obtained using the Spearman rank’s correlation test. Patient’s satisfaction has been analyzed using shift tables and the McNemar test. The analyses have been performed in the FAS population. No formal hypothesis testing of safety data (Adevrse Effect (AE) and drop-off) has been undertaken, but descriptive statistics have been performed. The Safety Analysis Set (SAS) has been used when summarizing safety data. Safety Set included all subjects who received at least one treatment cycle consisting of two injections at a 1 week interval.

## Results

### Study population

**Fig. 2:**
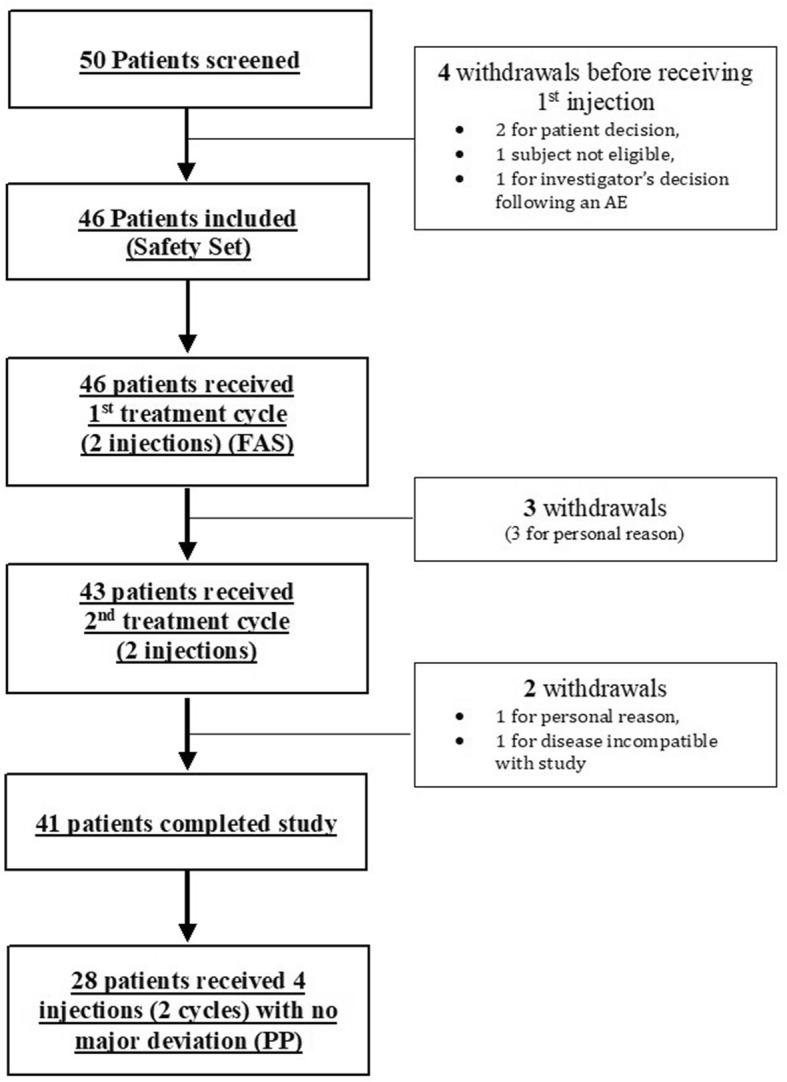
Patient disposition. FAS = Full Analysis Set; PP = Per Protocole

Fifty patients [39] were screened from which 46 entered the Multicenter Osteoarthritis Knee Hyaluronic Acid (MOKHA) Study. Forty-one (41; 89%) completed the study and 5 (11%) discontinued: 4 for personal reasons not related to the study and 1 for disease incompatible with the pursuing of the study decided by the patient. Eighteen (39.1%) patients out of 46 presented at least one major deviation. Major deviations were intake of prohibited medication (*n* = 10; 21.7%), no respect of the treatment regimen (*n* = 3; 6.5%), the presence of at least one exclusion criteria (*n* = 2; 4.3%) or the non-respect of delays between visits (*n* = 7; 15.2%). Forty-six patients received at least one treatment injection (SAS), 46 patients received at least one treatment cycle consisting of two injections at on week intervals (FAS) and 28 patients received a total dose of 4 injections without any major protocol violations (PP) (Fig. 2).

### Patient characteristics

Patients were mainly women (67.4%) with a mean age of 61.4 (9.6) years and a BMI of 30.60 [5] kg/m^2^. Patients were suffering from knee OA for a mean of 4.54 (5.9) years. The most painful knee was the right knee for 67.4% of the patients. Sixty-three (63) percents had a KL grading of II and 37% a KL of III (Table 2).

**Table 2:** Demographic data and OA history of the FAS population (*N* = 46)

Age (years; mean (SD)	61.4 (9.6)
Sex (%)	
*Women*	67.4%
*Men*	32.6%
BMI (Kg/m^2^; mean (SD))	30.6 (5)
Disease duration (years; mean (SD))	4.54 (5.9)
Evaluated knee (%)	
*Left*	*32.6%*
*Right*	*67.4%*
Radiological score (Kellgren &Lawrence)	
*Score II*	*63%*
*Score III*	*37%*

### Primary efficacy outcomes

sColl2–1 showed a significant increase at D90 and over in both FAS and PP population (Tables 3 and 4). sColl2–1 NO2 showed a significant increase only at D360 in the FAS (Table 3), but not in the PP population (Table 4). At D360, the effect size calculated on the FAS population was 0.47 (*p* = 0.022) for Coll2–1and 0.32 for Coll2–1NO2 (*p* = 0.048). uCTX-II Biomarker did not show any significant change over time in both FAS and PP population (Tables 3 and 4).

**Table 3:** levels (median (IQR)) of soluble biomarkers in the Full Analysis Set Population

	D0	D30	D90	D180	D210	D360
sColl2–1(nM)	*n* = 46; 498.66 (297.61;647.44)	*n* = 46; 578.30 (399.97;724.51)	*n* = 43; 634.46 (439.25;787.76)	*n* = 43; 635.01 (484.51;824.38)	*n* = 39; 622.47 (476.03;858.14)	*n* = 42;722.09 (469.99;883.08)
*Change from baseline*		*n = 46; 20.84 (−42.75;132.08)*	*n = 43; 50.25 (−42.03;242.73)*	*n = 43; 149.62 (28.17;297.19)*	*n = 39; 138.98 (−52.42;241.88)*	*n = 42;135.36 (−6.52;319.60)*
*P*-value vs D0		0.098	0.021	< 0.001	0.010	< 0.001
sColl2-NO2(pg/ml)	*n* = 30; 340.21 (266.24;425.99)	*n* = 36; 268.61 (206.13;370.75)	*n* = 34; 293.75 (233.64;464.92)	*n* = 34; 336.82 (265.68;450.43)	*n* = 29; 396.35 (284.85;598.79)	*n* = 31; 384.53 (303.84;663.99)
*Change from baseline*		*n = 30; −28.54 (−71.03;53.08)*	*n = 27; −17.33 (−64.21;67.82)*	*n = 26; 18.57 (−49.93;120.00)*	*n = 22; 29.31 (− 67.38;198.32)*	*n = 25; 69.75 (− 13.51;294.24)*
*P*-value vs D0		0.243	0.907	0.225	0.272	0.027
uCTX-II (ug/mmol creatinine)	*n* = 37; 0.012 (0.009;0.015)	*n* = 37; 0.012 (0.009;0.015)	*n* = 34; 0.010 (0.008;0.016)	*n* = 37; 0.012 (0.009;0.015)	*n* = 34; 0.011 (0.008;0.015)	*n* = 36; 0.012 (0.008;0.016)
*Change from baseline*		*n = 33; 0.001 (−0.002;0.003)*	*n = 31; 0.000 (−0.003;0.002)*	*n = 33; 0.000 (− 0.002;0.002)*	*n = 31; 0.000 (− 0.002;0.003)*	*n = 33; 0.000 (− 0.003;0.003)*
*P*-value vs D0		0.733	0.924	0.882	0.924	0.910
sPIINP (ng/ml)	*n* = 45; 599.50 (502.45; 812.42)	*n* = 42; 667.45 (519.13; 827.70)	*n* = 40; 708.90 (548.63; 873.46)	*n* = 41; 780.20 (572.88; 983.53)	*n* = 37; 858.79 (738.85;1061.52)	*n* = 37; 1057.41 (726.91;1268.47)
*Change from baseline*		*n = 41; 31.2 (−87.64;117.27)*	*n = 40; 74.82 (−83.03;193.95)*	*n = 41; 96.97 (5.27;298.16)*	*n = 37; 190.11 (138.91;319.81)*	*n = 37; 433.90 (136.71;573.78)*
*P*-value vs D0		0.954	0.038	< 0.001	< 0.001	< 0.001
sColl2–1(nM)/sPIINP (ng/ml)	*n* = 45; 0.72 (0.53; 0.96)	*n* = 42; 0.80 (0.56; 1.03)	*n* = 40; 0.69 (0.56; 1.05)	*n* = 41; 0.81 (0.62; 1.00)	*n* = 37; 0.69 (0.54;0.93)	*n* = 37; 0.56 (0.48;0.81)
*Change from baseline*		*n = 41; 0.04 (−0.11; 0.26)*	*n = 40; − 0.02 (− 0.18; 0.30)*	*n = 41; 0.04 (− 0.14; 0.36)*	*n = 37; − 0.04 (− 0.26; 0.22)*	*n = 37; − 0.11 (− 0.28; 0.00)*
*P*-value vs D0		0.291	0.677	0.257	0.678	0.005
uCTX-II (ng/mmol creatinine)/ sPIINP (ng/ml)	*n* = 36; 0.019 (0.012; 0.027)	*n* = 33; 0.018 (0.012; 0.026)	*n* = 33; 0.015 (0.011; 0.022)	*n* = 36; 0.016 (0.011; 0.024)	*n* = 33; 0.014 (0.011; 0.020)	*n* = 31; 0.012 (0.008; 0.019)
*Change from baseline*		*n = 28; −0.001 (− 0.004; 0.003)*	*n = 30; − 0.004 (− 0.008; 0.002)*	*n = 32; − 0.003 (− 0.008; 0.001)*	*n = 30; − 0.004 (− 0.010; − 0.001)*	*n = 28; − 0.006 (− 0.013; − 0.001)*
*P*-value vs D0		0.219	0.041	0.010	0.004	0.001
sCS-846 (ng/ml)	*n* = 38; 153.03 (124.60;186.18)	*n* = 38; 141.94 (125.27;185.74)	*n* = 36; 150.27 (114.88;180.88)	*n* = 37; 163.23 (134.32;185.58)	*n* = 36; 153.50 (121.16;178.61)	*n* = 39; 157.41 (130.60;186.27)
*Change from baseline*		*n = 34; −6.35 (−19.86;12.92)*	*n = 32; −5.52 (−24.24;19.69)*	*n = 31; −1.54 (− 18.28;22.55)*	*n = 30; − 1.97* (− 19.82;20.00)	*n = 33; 2.67* (− 17.60;19.17)
*P*-value vs D0		0.086	0.419	0.863	0.756	0.787
sCOMP (ng/ml)	*n* = 46; 796.15 (615.97;1044.40)	*n* = 45; 823.23 (617.95;1032.53)	*n* = 43; 737.14 (602.20;982.47)	*n* = 43; 740.58 (626.94;968.86)	*n* = 39; 763.79 (573.95;991.30)	*n* = 42; 846.46 (613.54;1060.12)
*Change from baseline*		*n = 45; −14.69 (−94.76;118.464)*	*n = 43; −4.86 (− 164.98;89.46)*	*n = 43; −26.32 (− 116.87;79.57)*	*n = 39; −24.32 (− 98.96;108.31)*	*n = 42; 15.30 (− 123.31;109.46)*
*P*-value vs D0		0.934	0.310	0.404	0.701	0.936
sMMP-3 (ng/ml)	*n* = 46; 14.86 (11.99;20.90)	*n* = 46; 15.75 (12.48;23.64)	*n* = 43; 16.36 (13.71;22.17)	*n* = 42; 15.87 (12.05;21.91)	*n* = 38; 15.95 (12.54;22.27)	*n* = 42; 14.87 (11.03;19.16)
*Change from baseline*		*n = 46; 1.19 (−1.11;3.17)*	*n = 43; 1.61 (− 1.37;3.53)*	*n = 42; 0.62 (−2.64;3.33)*	*n = 38; 0.85 (− 1.81;2.68)*	*n = 42; −0.03 (−3.21;2.57)*
*P*-value vs D0		0.138	0.041	0.726	0.420	0.792
sIL-6 (pg/ml)	*n* = 46; 1.62 (1.23;2.16)	*n* = 45; 1.70 (1.35;2.63)	*n* = 41; 1.67 (1.29;2.61)	*n* = 42; 1.54 (1.20;2.33)	*n* = 37; 1.71 (1.21;2.73)	*n* = 42; 1.95 (1.35;2.86)
*Change from baseline*		*n = 45; 0.19 (−0.23;0.51)*	*n = 41; 0.04 (− 0.30;-0.61)*	*n = 42; 0.014 (− 0.23;0.49)*	*n = 37; 0.175 (− 0.28;0.44)*	*n = 42; 0.26 (− 0.31;1.04)*
*P*-value vs D0		0.119	0.448	0.534	0.410	0.081
sMPO (ng/ml)	*n* = 44; 176.08 (128.28;251.10)	*n* = 44; 189.13 (104.79;260.37)	*n* = 41; 182.85 (132.41;288.12)	n = 41; 184.19 (105.37;264.12)	*n* = 35; 158.36 (87.61;250.25)	*n* = 38; 168.92 (11.21;239.39)
*Change from baseline*		*n = 43; −3.77 (−52.92;69.34)*	*n = 40; 1.32 (−45.19;75.32)*	*n = 39; 0.07 (−71.70;78.37)*	*n = 34; −24.03 (−64.88;39.60)*	*n = 36; − 11.28 (− 39.45;12.45)*
*P*-value vs D0		0.767	0.412	0.816	0.183	0.202

**Table 4:** levels (median (IQR)) of soluble biomarkers in the Per Protocol Population

	D0	D30	D90	D180	D210	D360
sColl2–1(nM)	*n* = 28; 462.04 (297.03;604.49)	*n* = 28; 556.76 (399.84;692.02)	*n* = 28; 653.36 (429.74;757.79)	*n* = 28; 599.67 (456.70;725.51)	*n* = 24; 557.73 (460.48;818.09)	*n* = 27; 598.44 (408.51;858.57)
*Change from baseline*		*n = 28; 37.03 (−45.30;140.52)*	*n = 28; 61.29 (−31.90;250.43)*	*n = 28; 106.88 (31.31;265.17)*	*n = 24; 145.72 (−40.33;201.72)*	*n = 27; 123.74 (−6.52;273.51)*
*P*-value vs D0		0.162	0.018	0.001	0.066	0.005
sColl2-NO2 (pg/ml)	*n* = 20; 335.13 (268.79;467.17)	*n* = 23; 271.58 (222.80;393.76)	*n* = 23; 283.65 (212.03;599.42)	*n* = 22; 317.89 (264.34;450.43)	*n* = 18; 491.21 (271.25;624.31)	*n* = 20; 485.38 (307.04;740.40)
*Change from baseline*		*n = 20; −34.88 (−65.31;62.77)*	*n = 18; −36.62 (−73.09;87.12)*	*n = 18; −9.71 (− 65.55;104.32)*	*n = 14; 44.37 (− 67.38;202.55)*	*n = 16; 76.89 (−55.92;334.31)*
*P*-value vs D0		0.498	0.832	0.899	0.426	0.175
uCTX-II (ug/mmol creatinine)	*n* = 24; 0.012 (0.008;0.014)	*n* = 25; 0.012 (0.009;0.015)	*n* = 23; 0.010 (0.008;0.016)	*n* = 26; 0.012 (0.009;0.014)	*n* = 23; 0.011 (0.007;0.015)	*n* = 23; 0.010 (0.008;0.014)
*Change from baseline*		*n = 23; 0.001 (−0.002;0.003)*	*n = 21; 0.001 (−0.003;0.001)*	*n = 23; 0.000 (− 0.003;0.003)*	*n = 21; 0.000 (− 0.002;0.003)*	*n = 22; − 0.001 (− 0.003;0.002)*
*P*-value vs D0		0.357	0.626	0.791	0.987	0.420
sPIINP (ng/ml)	*n* = 27; 590.14 (500.89; 740.87)	*n* = 24; 651.57 (529.91; 875.12)	*n* = 26; 697.9 (544.21; 858.85)	*n* = 27; 780.2 (572.88; 999.99)	*n* = 22; 865.69 (752.23;1075.72)	*n* = 24; 1002.69 (664.39;1263.72)
*Change from baseline*		*n = 23; 5.83 (−87.64;87.38)*	*n = 26; 57.68 (− 96.03;172.51)*	*n = 27; 90.35 (−23.12;298.16)*	*n = 22; 198.77 (144.54;368.52)*	*n = 24; 417.05 (115.26;529.05)*
*P*-value vs D0		0.791	0.434	0.004	< 0.001	< 0.001
sColl2–1 (nM)/sPIINP (ng/ml)	*n* = 27; 0.66 (0.49; 0.96)	*n* = 24; 0.69 (0.45; 0.93)	*n* = 26; 0.78 (0.60; 1.00)	*n* = 27; 0.76 (0.60; 0.94)	*n* = 22; 0.61 (0.54;0.82)	*n* = 24; 0.52 (0.44;0.62)
*Change from baseline*		*n = 23; 0.07 (−0.09; 0.24)*	*n = 26; 0.00 (− 0.08; 0.34)*	*n = 27; 0.04 (− 0.21; 0.20)*	*n = 22; − 0.04 (− 0.40; 0.12)*	*n = 24; − 0.12 (− 0.30; − 0.01)*
*P*-value vs D0		0.465	0.258	0.665	0.306	0.022
uCTX-II (ng/mmol creatinine)/ sPIINP (ng/ml)	*n* = 23; 0.018 (0.010; 0.028)	*n* = 21; 0.025 (0.011; 0.029)	*n* = 22; 0.015 (0.010; 0.018)	*n* = 25; 0.016 (0.011; 0.024)	*n* = 22; 0.012 (0.008; 0.016)	*n* = 20; 0.011 (0.007; 0.019)
*Change from baseline*		*n = 18; −0.001 (− 0.004; 0.003)*	*n = 20; − 0.002 (− 0.007; 0.002)*	*n = 22; − 0.004 (− 0.008; 0.002)*	*n = 20; − 0.003 (− 0.011; − 0.001)*	*n = 19; −0.007 (− 0.013; − 0.002)*
*P*-value vs D0		0.777	0.218	0.046	< 0.001	< 0.001
sCS-846 (ng/ml)	*n* = 23; 140.14 (117.75;186.18)	*n* = 23; 127.73 (123.57;188.92)	*n* = 24; 131.47 (113.90;174.13)	*n* = 24; 156.04 (127.58;183.30)	*n* = 23; 161.33 (120.75;174.33)	*n* = 25; 164.94 (135.04;204.99)
*Change from baseline*		*n = 21; − 1.82 (− 17.50;5.25)*	*n = 21; −7.30 (−23.49;7.85)*	*n = 20; 13.64 (−5.99;20.73)*	*n = 19; 1.534* (− 15.11;29.84)	*n = 21; 11.30* (−4.93;24.80)
*P*-value vs D0		0.352	0.212	0.231	0.651	0.143
sCOMP (ng/ml)	*n* = 28; 802.85 (613.44;1050.03)	*n* = 28; 855.56 (607.44;1097.99)	*n* = 28; 746.93 (611.46;979.58)	*n* = 28; 751.8 (617.94;977.22)	*n* = 24; 770.23 (584.69;1014.59)	*n* = 27; 800.09 (613.54;1060.12)
*AChange from baseline*		*n = 28; 26.33 (−43.24;152.94)*	*n = 28; 5.75 (−107.05;107.18)*	*n = 28; − 10.06 (− 109.63;121.00)*	*n = 24; − 33.70 (− 113.39;103.32)*	*n = 27; 13.56 (− 133.86;109.46)*
*P*-value vs D0		0.176	0.929	0.947	0.678	0.797
sMMP-3 (ng/ml)	*n* = 28; 14.67 (11.89;21.58)	*n* = 28; 16.43 (12.14;23.93)	*n* = 28; 15.98 (13.92;22.67)	*n* = 28; 16.00 (12.23;22.15)	*n* = 24; 16.53 (12.9;23.0)	*n* = 27; 15.63 (11.56;24.06)
*Change from baseline*		*n = 28; 1.19 (−0.91;2.97)*	*n = 28; 1.94 (− 0.19;4.29)*	*n = 28; 0.91 (−2.52;3.48)*	*n = 24; 1.39 (− 0.92;2.7)*	*n = 27; 0.46 (−1.99;3.58)*
*P*-value vs D0		0.282	0.006	0.348	0.071	0.511
sIL-6 (pg/ml)	*n* = 28; 1.47 (1.10;2.00)	*n* = 27; 1.60 (1.22;2.35)	*n* = 27; 1.67 (1.28;2.42)	*n* = 28; 1.49 (1.11;2.47)	*n* = 23; 1.48 (1.13;2.23)	*n* = 27; 1.83 (0.97;2.86)
*Change from baseline*		*n = 27; 0.21 (−0.12;0.48)*	*n = 27; 0.03 (− 0.30;-0.61)*	*n = 28; − 0.004 (− 0.11;0.49)*	*n = 23; 0.21 (− 0.09;0.46)*	*n = 27; 0.33 (− 0.13;1.13)*
*P*-value vs D0		0.030	0.481	0.348	0.108	0.017
sMPO (ng/ml)	*n* = 27; 199.49 (140.46;264.05)	*n* = 28; 240.45 (134.93;308.59)	*n* = 28; 187.17 (142.02;336.18)	*n* = 28; 208.81 (114.126;285.47)	*n* = 22; 161.96 (82.81;241.45)	*n* = 24; 174.51 (93.23;216.50)
*Change from baseline*		*n = 27; 36.24 (−41.33;96.54)*	*n = 27; 30.40 (−48.99;84.18)*	*n = 27; 22.83 (−71.70;95.12)*	*n = 21; −16.46 (−62.60;39.60)*	*n = 23; − 4.74 (− 43.30;24.41)*
*P*-value vs D0		0.160	0.237	0.481	0.317	0.555

### Secondary efficacy outcomes

#### Biological parameters

Among all biomarkers tested as secondary outcomes, only sPIIANP levels significantly changed during the study (Tables 3 and 4). A significant increase of sPIIANP was observed from D90 in the FAS population and from D180 in the PP set. The other biomarkers did not vary overtime.

Further, the increase of PIIANP between D0 and D90 was significantly lower in OARSI-OMERAC responders than in non-responders (responders 39.3157.43 (208–84.65; 172.50) ng/ml vs non-responders 239.41258.27 (46.09182.03; 273.51) ng/ml; *p* = 0.008). No difference was observed if we consider the variation of PIIANP between D0 and D360.

No significant differences at baseline or changes over time were observed in sColl2-1, Coll2–1 N02 or CTX-II levels between clinical responders and non-responders. The ratios Coll2–1/PIIANP at D360 (*p* = 0.005) and CTX-II/PIIANP from D90 to D360 (0.05 < *p* < 0.001) decreased, indicating a decrease of cartilage catabolism. At D360, the effect size calculated on the FAS population was 0.46 (*p* = 0.005) and 0.720 (*p* = 0.001) for the ratio Coll2–1/PIIANP and CTX-II/PIIANP respectively.

#### MRI features

WORMS total score, that represents a summation of grades for all of the knee features, showed a significant (*p* = 0.037) increase at D360. WORMS total cartilage and total cysts features were also significantly increased (cartilage: mean change (SD): 0.45 (1.21); *p* = 0.025); cysts: mean change (SD): 0.23 (0.63), *p* = 0.047) at D360. Interestingly, WORMS effusion score was significantly decreased at D180 that was not maintained at D360. Other WORMS total features or WORMS compartments were not significantly modified over time (Table 5).

**Table 5:** WORMS total score and by features (mean (SD)) in the Full Analysis Set population

	D0	D180	D360
WORMS total score	63.95 (27.78)	64.39 (27.71)	64.08 (28.03)
*Change from baseline*		*0.38 (1.77)*	*0.96 (2.75)*
*p* value		0.183	0.037
Cartilage	23.83 (11.27)	23.01 (11.13)	23.03 (11.45)
*Change from baseline*		*0.18 (0.70)*	*0.45 (1.21)*
*p* value		0.188	0.025
Cyst	2.73 (2.65)	2.83 (2.65)	2.90 (2.62)
*Change from baseline*		0.10 (0.30)	0.23 (0.63)
*p value*		0.125	0.047
Effusion	0.93 (0.69)	0.76 (0.54)	0.77 (0.54)
*Change from baseline*		*−0.17 (0.38)*	*−0.15 (0.54)*
*p value*		0.016	0.148

Mean T2 relaxation time (ms) was significantly decreased overtime in the femur compartment for 8 distinct features (Table 6): femur whole layers (median change: − 0.537 ms, *p* = 0.050 at D180 and − 1.595 ms, *p* = 0.014 at D360), femur middle layer (median change: − 0.596 ms, *p* = 0.03 at D180 and − 1.249 ms, *p* = 0.008 at D360), femur bottom layer (median change: − 2.051 ms, *p* = 0.009 at D360), lateral weight bearing femur whole (median change: − 3.129 ms, *p* = 0.011 at D180 and − 1.748 ms, *p* = 0.032 at D360), lateral weight bearing femur middle (median change: − 1.075 ms, *p* = 0.005 at D180 and − 1.264 ms, *p* = 0.045 at D360), lateral weight bearing femur bottom (median change: − 1.599 ms, *p* = 0.015 at D180). In contrast, mean T2 relaxation time was not modified in the medial compartment. Standard deviation of T2 relaxation was significantly reduced in the femoral cartilage for the 5 following distinct features: femur bottom (median change: − 0.944 ms, *p* = 0.027 at D180), lateral weight bearing femur bottom (median change: − 1.053 ms, *p* = 0.005 at D180), medial weight bearing femur middle (median change: − 1.355 ms, *p* = 0.037 at D180), medial weight bearing femur bottom (median change: − 1.506 ms, *p* = 0.010 at D180 and median change: − 2.298 ms, *p* = 0.012 at D360). Similarly, at patella level, mean and SD of T2 relaxation time showed a significant decrease, the majority being observed starting from D180, for 7 distinct features: patella medial trochlea whole, patella medial trochlea top, patella medial trochlea middle, patella medial trochlea bottom, patella lateral trochlea whole, patella lateral trochlea middle, patella lateral trochlea bottom. No significant changes were observed at the tibia level (Table 6).

**Table 6:** Mean T2 relaxation time and standard deviation of relaxation time for patella (median (IQR)) in the Full Analysis Set population

		Mean T2 relaxation time (ms)		SD of relaxation time (ms)	
		Median (IQR) *n* = 38	*p*-value	Median (IQR)*n* = 38	*p*-value
Whole Patella- *whole layer*	*D180* vs *D0* *D360* vs *D0*	2.822 (− 3.872; 8.996) 1.168 (− 7.721; 9.410)	0.403 0.593	0.627 (−3.895; 7.427) 0.236 (− 9.654; 7.812)	0.480 0.960
Whole Patella- *top layer*	*D180* vs *D0* *D360* vs *D0*	4.446 (−6.977; 16.697) 4.937 (− 10.051; 21.672)	0.320 0.327	6.644 (− 21.994; 20.796) 1.413 (− 16.726; 28.354)	0.593 0.603
Whole Patella- *middle layer*	*D180* vs *D0* *D360* vs *D0*	1.236 (−2.881; 6.889) 0.125 (− 6.598; 7.365)	0.356 0.803	0.788 (− 4.667; 6.221) − 0.333 (− 11.132; 13.976)	0.603 0.870
Whole Patella- *bottom layer*	*D180* vs *D0* *D360* vs *D0*	0.202 (− 3.239; 5.418) − 0.228 (− 2.205; 5.368)	0.545 0.633	1.115 (− 2.416; 5.700) 0.408 (− 4.172; 6.304)	0.320 0.644
Medial trochlea - *whole layer*	*D180* vs *D0* *D360* vs *D0*	−2.354 (− 16.342; 4.102) − 2.429 (− 12.540; 0.968)	0.061 0.019	−1.959 (− 8.472; 1.353) − 0.818 (− 6.424; 1.126)	0.008 0.107
Medial trochlea - *top layer*	*D180* vs *D0* *D360* vs *D0*	−1.768 (− 8.476; 1.722) − 1.777 (− 9.093; 2.549)	0.027 0.075	−1.774 (− 6.983; 1.804) − 0.449 (− 5.186; 1.711)	0.023 0.229
Medial trochlea - *middle layer*	*D180* vs *D0* *D360* vs *D0*	1.606 (− 14.664; 2.549) − 1.45 (− 10.896; 0.991)	0.032 0.024	−2.074 (− 9.846; 2.389) − 0.796 (− 7.151; 0.737)	0.011 0.033
Medial trochlea- *bottom layer*	*D180* vs *D0* *D360* vs *D0*	−2.303 (− 21.865; 7.574) − 1.852 (− 16.764; 0.791)	0.16 0.023	−2.703 (− 13.902; 2.017) − 1.574 (− 8.096; 2.390)	0.006 0.124
Lateral trochlea - *whole layer*	*D180* vs *D0* *D360* vs *D0*	− 2.167 (− 10.398; 0.720) − 2.107 (− 11.110; 0.938)	0.001 0.006	− 0.804 (− 6.646; 2.799) − 0.508 (− 3.996; 0.839)	0.110 0.117
Lateral trochlea - *top layer*	*D180* vs *D0* *D360* vs *D0*	− 0.410 (− 8.520; 4.261) 0.169 (− 5.191; 7.014)	0.623 0.848	1.069 (− 7.704; 10.070) 0.670 (− 3.756; 8.526)	0.972 0.387
Lateral trochlea - *middle layer*	*D180* vs *D0* *D360* vs *D0*	−3.663 (− 11.111; 0.482) − 2.380 (− 10.532; 0.399)	0.001 0.001	− 1.473 (− 9.228; 0.898) − 1.547 (− 7.190; − 0.374)	0.009 0.003
Lateral trochlea - *bottom layer*	*D180* vs *D0* *D360* vs *D0*	−2.538 (− 26.748; 0.497) − 2.677 (− 20.622; 0.621)	0.001 0.002	−1.188 (− 8.698; 0.999) − 2.245 (− 9.955; 1.961)	0.005 0.012

Cartilage volume significantly increased at D360 in the lateral weight bearing femur (median change = 66 mm^3^, *p* = 0.03) and the patella lateral trochlea (median change = 77 mm^3^, *p* = 0.028). No significant volume change was observed in the other compartments.

Cartilage thickness significantly increased at D360 in the lateral weight bearing femur (median change: 0.023 mm). No change was observed in the other compartments (Table 7).

**Table 7:** Mean T2 relaxation time and standard deviation of relaxation time for femur (median (IQR)) in the Full Analysis Set population

		Mean T2 relaxation time (ms)		SD of relaxation time (ms)	
		Median (IQR) *n* = 38	*p*-value	Median (IQR) *n* = 38	*p*-value
Whole Femur- *whole layer*	*D180* vs *D0* *D360* vs *D0*	−0.537 (−10.658; 2.368) − 1.595 (−9.338; 1.532)	0.050 0.014	−1.315 (−4.361; 2.227) − 0.588 (− 4.128; 1.634)	0.160 0.279
Whole Femur- *top layer*	*D180* vs *D0* *D360* vs *D0*	− 1.589 (−6.682; 2.216) − 1.678 (− 6.797; 3.763)	0.080 0.131	− 0.697 (− 7.715; 5.048) 1.159 (− 5.690; 6.124)	0.574 0.545
Whole Femur- *middle layer*	*D180* vs *D0* *D360* vs *D0*	−0.596 (10.378; 1.938) − 1.249 (− 10.187; 0.967)	0.030 0.008	−1.524 (− 5.920; 1.637) − 0.305 (− 5.309; 1.955)	0.86 0.174
Whole Femur- *bottom layer*	*D180* vs *D0* *D360* vs *D0*	−0.259 (− 10.454; 3.226) − 2.051 (− 10.495; 1.684)	0.094 0.009	−0.944 (− 8.002; 1.725) − 0.402 (− 8.032; 1.057)	0.027 0.055
Medial weight bearing femur- *whole layer*	*D180* vs *D0* *D360* vs *D0*	0.318 (− 14.381; 3.036) − 1.680 (− 13.240; 2.583)	0.198 0.147	−1.320 (− 6.054; 2.027) − 0.585 (− 5.550; 1.085)	0.059 0.124
Medial weight bearing femur- *top layer*	*D180* vs *D0* *D360* vs *D0*	−1.119 (− 8.54; 3.575) − 1.923 (− 7.076; 4.147)	0.219 0.480	0.493 (−4.423; 3.233) − 1.741 (− 3.532; 10.667)	0.782 0.717
Medial weight bearing femur- *middle layer*	*D180* vs *D0* *D360* vs *D0*	0.115 (− 12.221; 2.005) − 1.725 (− 8.003; 0.934)	0.086 0.057	−1.335 (− 8.136; 1.699) − 1.718 (− 6.450; 0.685)	0.037 0.050
Medial weight bearing femur- *bottom layer*	*D180* vs *D0* *D360* vs *D0*	−0.261 (− 13.971; 5.085) − 2.727 (− 10.766; 3.163)	0.254 0.053	−1.506 (− 11.723; 1.387) − 2.298 (− 8.391; 0.853)	0.010 0.012
Lateral weight bearing femur- *whole layer*	*D180* vs *D0* *D360* vs *D0*	−3.129 (− 8.423; 1.453) − 1.748 (− 8.187; 2.160)	0.011 0.032	0.179 (− 3.713; 2.078) − 0.123 (− 3.475; 2.057)	0.613 0.613
Lateral weight bearing femur- *top layer*	*D180* vs *D0* *D360* vs *D0*	0.580 (−7.286; 4.058) − 0.628 (− 6.597; 3.953)	0.664 0.364	−0.375 (− 11.374; 5.426) 0.897 (− 5.299; 6.409)	0.412 0.792
Lateral weight bearing femur- *middle layer*	*D180* vs *D0* *D360* vs *D0*	−1.075 (− 7.777; 1.477) − 1.264 (− 9.870; 1.896)	0.005 0.045	0.183 (− 3.536; 1.522) − 0.589 (− 3.179; 1.625)	0.395 0.349
Lateral weight bearing femur- *bottom layer*	*D180* vs *D0* *D360* vs *D0*	−1.599 (− 12.514; 2.41) − 2.164 (− 10.944; 3.286)	0.015 0.100	−1.053 (− 5.959; 0.667) − 0.699 (− 6.740; 1.931)	0.005 0.110

Neutral, positive and negative curvature correspond to a flat, convex, and concave surface respectively. Cartilage curvature significantly decreased at D360 for 3 distinct convex (positive curvature) surfaces: femur (− 0,001 mm^− 1^, *p* = 0.015), medial weight bearing femur (− 0,001 mm^− 1^, *p* = 0.027), lateral tibia (− 0,002 mm^− 1^, *p* = 0.02). These results suggest a flattening of medial femur shape.

#### Pain and function

**Fig. 3:**
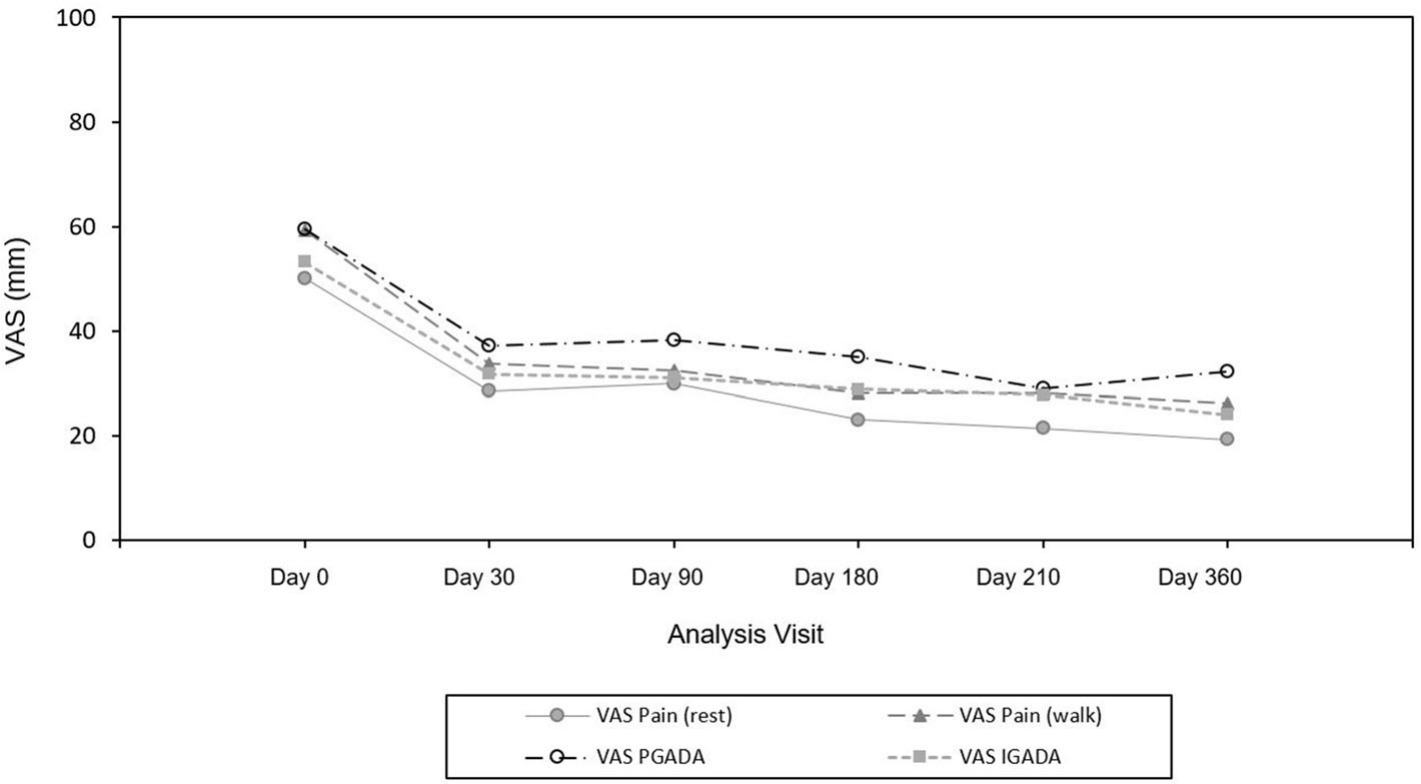
Time evolution of pain at rest, at walking, and patients and investigators global assessment. VAS = Visual Analog scale; PGADA = Patient Global Assessment of Disease Activity; IGADA = Investigator Global Assessment of Disease Activity

In comparison to baseline, VAS at rest and while walking was significantly decreased at each time point (mean decrease from − 20.1 mm to − 29.9 mm and from − 25.5 mm to − 31.5 mm at rest and while walking respectively, *p* < 0.001) (Fig. 3). Similarly, patient and investigator global assessment of the disease activity scores were significantly reduced at each time point compared to baseline value (*p* < 0.001) (Fig. 3, Table 8).

**Table 8:** VAS changes from baseline (mean (SD)) in the Full Analysis Set Population

VAS (mm)	D30 vs D0	D90 vs D0	D180 vs D0	D210 vs D0	D360 vsD0
Knee pain at rest	*n* = 46 −21.7 (23.7)	*n* = 43 − 20.1 (24.5)	*n* = 43 − 26.9 (25.1)	*n* = 39 − 29.9 (24.4)	*n* = 41 − 29.8 (23.8)
*P*-value	< 0.001	< 0.001	< 0.001	< 0.001	< 0.001
Knee pain while walking	*n* = 45 − 25.5 (24.0)	*n* = 42 − 26.9 (24.7)	*n* = 42 − 30.2 (29.4)	*n* = 39 − 31.3 (31.1)	*n* = 40 − 31.5 (31.5)
*P*-value	< 0.001	< 0.001	< 0.001	< 0.001	< 0.001
Patient’s global assessment of disease activity	*n* = 45 − 22.7 (27.8)	*n* = 42 − 21.0 (26.4)	*n* = 42 − 23.9 (28.5)	*n* = 39 − 30.5 (30.6)	*n* = 40 − 25.3 (31.5)
*P*-value	< 0.001	< 0.001	< 0.001	< 0.001	< 0.001
Investigator global assessment of disease activity	*n* = 42 − 21.0 (22.3)	*n* = 40 − 21.4 (25.0)	*n* = 41 − 23.0 (24.9)	*n* = 37 − 24.4 (23.9)	*n* = 37 − 25.8 (25.5)
*P*-value	< 0.001	< 0.001	< 0.001	< 0.001	< 0.001

**Fig. 4:**
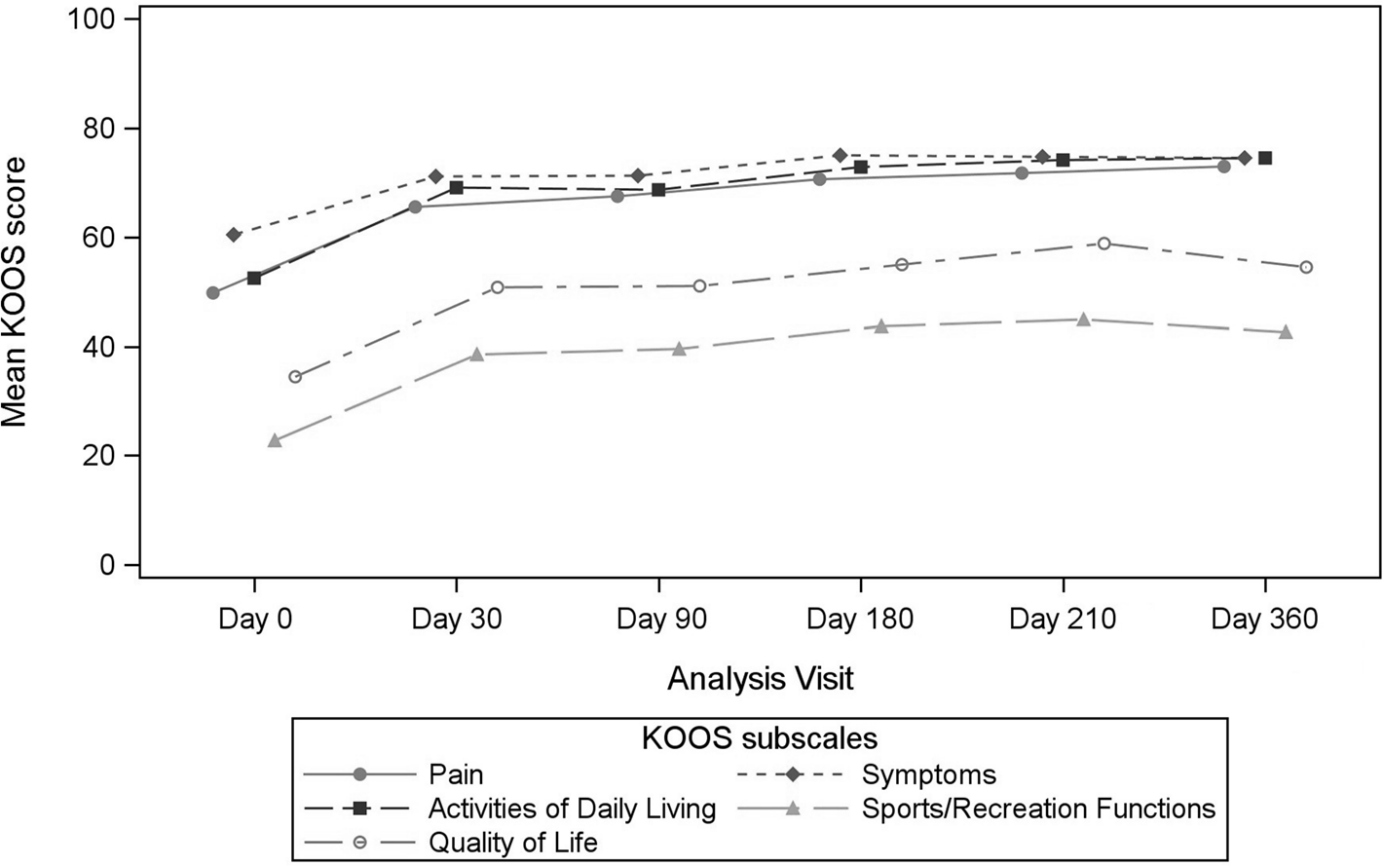
Time evolution of Knee Injury and Osteoarthritis Outcome (KOOS) global score and subscores

KOOS global score and subscale significantly increased overtime (mean improvement from 16.173 to 21.341 and from 15.384 to 20.511 in pain sub-score and in activities of daily living score respectively; *p* < 0.001 for all sub-scales) (Fig. 4, Table 9). The ratio of OARSI-OMERACT responders in the FAS population increased overtime. This ratio was 48,9% at D180 and 70.7% at D360.

**Table 9:** KOOS changes from baseline (mean (SD)) in the Full Analysis Set Population

KOOS scores	D30 vs D0	D90 vs D0	D180 vs D0	D210 vs D0	D360 vsD0
Pains	*n* = 45 16.173 (18.13)	*n* = 42 16.968 (18.36)	*n* = 42 20.106 (21.62)	*n* = 39 20.664 (20.22)	*n* = 41 21.341 (18.68)
*P*-value	< 0.001	< 0.001	< 0.001	< 0.001	< 0.001
Symptoms	*n* = 45 11.164 (16.04)	*n* = 42 10.431 (17.05)	*n* = 42 13.747 (14.59)	*n* = 39 12.790 (16.27)	*n* = 41 14.184 (17.27)
*P*-value	< 0.001	< 0.001	< 0.001	< 0.001	< 0.001
Activity of daily living	*n* = 45 16.97 (16.08)	*n* = 42 15.384 (18.49)	*n* = 42 19.60 (20.80)	*n* = 39 19.92 (17.72)	*n* = 41 20.51 (16.82)
*P*-value	< 0.001	< 0.001	< 0.001	< 0.001	< 0.001
Sports/ Recreation function	*n* = 45 15.963 (19.21)	*n* = 41 17.31 (27.40)	*n* = 40 18.65 (28.17)	*n* = 38 21.930 (26.79)	*n* = 40 17.563 (24.17)
*P*-value	< 0.001	< 0.001	< 0.001	< 0.001	< 0.001
Quality of life	*n* = 45 16.898 (26.82)	*n* = 42 15.774 (25.43)	*n* = 42 19.643 (26.54)	*n* = 39 23.077 (26.45)	*n* = 41 18.902 (29.23)
*P*-value	< 0.001	< 0.001	< 0.001	< 0.001	< 0.001
KOOS total	*n* = 45 77.171 (76.99)	*n* = 42 73.368 (85.49)	*n* = 42 90.854 (88.37)	*n* = 39 97.820 (85.82)	*n* = 41 91.95 (83.47)
*P*-value	< 0.001	< 0.001	< 0.001	< 0.001	< 0.001

#### Patient’s satisfaction

At D30, patient satisfaction was good, with 43.5% of patients feeling better, 37% feeling a little better, 15.2% feeling no change, 4.3% feeling little lower and none far lower. Between D30 and D360, patient’s satisfaction remained stable.

### Correlations between imaging, clinical and biological parameters

At baseline, the VAS walking pain were positively and significantly correlated with cartilage volume (r = − 0.42779; *p* = 0.007) and thickness (r = − 0.38758; *p* = 0.015). VAS changes overtime (from baseline to D180 or from baseline to D360) were not correlated with MRI changes overtime.

At baseline, VAS walking pain was positively correlated with sColl2–1NO2 levels (r = − 0.53617; *p* = 0.002), but not with the other biomarkers. No significant correlation was found between VAS walking pain and biomarkers at follow-up time points.

### Safety

Ninety-five (95) AEs were reported after the first treatment cycle in 37 patients (80% of the SAS). Eleven patients (23.9%) presented an Adverse Device Reaction (ADR). In total, 15 ADRs (16% of AE) have been reported: difficulty to extend leg, redness of the patella, knee pain after injection, knee swelling, injection site pain, pain at injection, dizziness and knee swelling. Importantly, only one AE led to treatment discontinuation and only one led to patient withdrawal but were not related to the product. Eight (15.2% of the safety population) SAEs have been recorded during the study. Importantly, no SAE was linked to the study medication.

## Discussion

This non-controlled prospective study provided a lot of precious information on the evolution of biological, structural and clinical parameters after intra-articular injection of HYMOVIS®. These results will be valuable for the design of a randomized and controlled study aiming to demonstrate the disease modifying effect of viscosupplementation. We have investigated the evolution profile of biomarkers of type II collagen metabolism. Coll2–1 and CTX-II are both biomarkers of cartilage degradation [40–43] while PIIANP is a biomarker of collagen synthesis [44]. Interestingly, Coll2–1 and PIIANP increased overtime while CTX-II remained stable. As the ratio Coll2–1/PIIANP and CTX-II/PIIANP decreased overtime, these biomarkers variations can be interpreted as a positive effect of the HYMOVIS® on type II collagen turnover. However, in the absence of a control group, we can not exclude that these changes are simply the consequence of changes in the cartilage matrix turnover linked to natural disease evolution. The observation that Coll2–1 increased while CTX-II remained stable is surprising. This can be explained in two ways. First, CTX-II and Coll2–1 epitopes are generated by different mechanisms. CTX-II is a neoepitope located in the C-terminal telopeptide and generated enzymatically whereas Coll2–1 is a peptide located in triple helix and released after denaturation of the collagen [45]. Secondly, there is evidence that urinary level of CTX-II is influenced by bone remodeling which is not demonstrated for Coll2–1 [46, 47]. Therefore, we can hypothesize that metabolic change induced by viscosupplementation in a single joint is not sufficient to influence the urinary CTX-II level resulting from bone and cartilage remodeling, while as demonstrated previously, Coll2–1 is sensitive to changes occurring in one single joint [42, 48].

To demonstrate the structure-modifying effect of viscosupplementation, the EUROVISCO group recommends a combination of imaging and biological outcome measures [49]. A decrease of soluble biomarkers of cartilage degradation over time does not prove the chondroprotective effect of the treatment if this effect is not complemented by the imaging examinations. One strength of this study is that we have combined MRI and biological markers. Based on the literature review, a worsening of the MRI features was anticipated after 12 months follow-up [50]. In this study, T2 relaxation time analysis revealed an improvement of cartilage quality in total femur and in the lateral weight bearing femur as well as in the patella as soon as 6 months. WORMS effusion score, an indicator of synovitis, also revealed an improvement after 6 months. Finally, cartilage volume and thickness showed an increase in lateral weight bearing femur. The improvement in cartilage T2 values and volume in the lateral weight bearing femur can be interpreted as a beneficial effect of HYMOVIS®. These changes in cartilage structure and volume could be the result of type II collagen turnover increase as suggested by collagen-derived biomarkers. However, in this study, no significant correlation has been found between soluble biomarkers levels at baseline and the severity or changes of MRI features. Therefore, this explanation remains hypothetical.

A significant decrease of knee pain and an improvement of joint function was observed. Similarly, a significant improvement of patient and investigator global assessement was observed. These improvements are confirmed by the number of responders to treatment which increases over time. However, in the absence of control group, the interpretation of these improvements may be less credible since placebo is known to be effective at relieving pain and at improving function and stiffness [39, 51]. None of the biomarkers were enable to discriminate between OARSI-OMERACT non-responders and responders to treatments. This is probably due to the small sample size.

HYMOVIS® shows a good safety profile with no SAE related to the product. Remarkably, less than 15% of patients had definite product related reaction the marjority being pain and swelling at site injection and only one led to patient withdrawal. We can conclude that the product is well tolerated.

## Conclusion

This prospective study indicates that HYMOVIS®, a well-tolerated intra-articular treatment, significantly enhanced type II collagen turnover as suggested by the increase in Coll2–1 and PIIANP levels and cartilage volume observed by MRI in lateral knee compartment only. Importantly, this study highlighted the potential symptomatic benefit of HYMOVIS® on pain and function. Altogether, theses data suggest that HYMOVIS® could have a protective effect on cartilage and provides critical information for the design of a larger phase III clinical trial.

### Acknowlegments

HY is the coordinator of the WP1 of the D-BOARD Consortium funded by European Commission Framework 7 program (EU FP7; HEALTH.2012.2.4.5–2, project number 305815, Novel Diagnostics and Biomarkers for Early Identification of Chronic Inflammatory Joint Diseases) and principal investigator of the Excellence of Science project. Joint-T-against Osteoarthritis 30,480,119 funded by the elgian FWO/FNRS.

## Data Availability

The datasets used and/or analysed during the current study are available from the corresponding author on reasonable request.

## Abbreviations

ADR: Adverse Device Reaction
AE: Adverse Effect
AGG: Aggrecan
BMI: Body Mass Index
COMP: Cartilage Oligomeric Matrix Protein
DO: Drop-Off
FAS: Full Analysis Set
GCLP: Good Clinical Laboratory Practice
GCP: Good Clinical Practice
HA: Hyaluronic Acid
IA: Intra-Articular
IL: InterLeukin
KL: Kelgren and Lawrence
KOOS: Knee Injury and Osteoarthritis Outcome Score
MMP: Matrix Metalloprotease
MOKHA: Multicenter Osteoarthritis Knee Hyaluronic Acid
MPO: MyeloPerOxidase
MRI: Magnetic Resonance Imaging
MW: Molecular Weight
NSAIDS: Non-Steroidal Anti-Inflammatory Drugs
PP: Per-Protocol
PTH: ParaTHormone
RR: Responder rate
SAS: Safety Analysis Set
SD: Standard Deviation
SERM: Selective Estrogen-Receptor Modulator
SYSADOA: Symptomatic Slow Acting Drugs in OA
TKR: Total Knee Replacement
VAS: Visual Analog Scale
WORMS: Whole-Organ Magnetic Resonance Imaging Score
